# Sex differences in life history drive evolutionary transitions among maternal, paternal, and bi-parental care

**DOI:** 10.1002/ece3.494

**Published:** 2013-02-20

**Authors:** Hope Klug, Michael B Bonsall, Suzanne H Alonzo

**Affiliations:** 1 Department of Biological & Environmental Sciences, University of Tennessee–ChattanoogaDept. 2653, 615 McCallie Aven, Chattanooga, TN 37403, USA; 2 Department of Ecology & Evolutionary Biology, Yale UniversityPO Box 208106, 165 Prospect St, New Haven, CT 06520, USA; 3 Mathematical Ecology Research Group, Department of Zoology, University of OxfordOxford, OX1 3PS, UK

**Keywords:** Biparental care, invasion analysis, life-history, maternal care, parental care, paternal care

## Abstract

Evolutionary transitions among maternal, paternal, and bi-parental care have been common in many animal groups. We use a mathematical model to examine the effect of male and female life-history characteristics (stage-specific maturation and mortality) on evolutionary transitions among maternal, paternal, and bi-parental care. When males and females are relatively similar – that is, when females initially invest relatively little into eggs and both sexes have similar mortality and maturation – transitions among different patterns of care are unlikely to be strongly favored. As males and females become more different, transitions are more likely. If females initially invest heavily into eggs and this reduces their expected future reproductive success, transitions to increased maternal care (paternal → maternal, paternal → bi-parental, bi-parental → maternal) are favored. This effect of anisogamy (i.e., the fact that females initially invest more into each individual zygote than males) might help explain the predominance of maternal care in nature and differs from previous work that found no effect of anisogamy on the origin of different sex-specific patterns of care from an ancestral state of no care. When male mortality is high or male egg maturation rate is low, males have reduced future reproductive potential and transitions to increased paternal care (maternal → paternal, bi-parental → paternal, maternal → bi-parental) are favored. Offspring need (i.e., low offspring survival in the absence of care) also plays a role in transitions to paternal care. In general, basic life-history differences between the sexes can drive evolutionary transitions among different sex-specific patterns of care. The finding that simple life-history differences can alone lead to transitions among maternal and paternal care suggests that the effect of inter-sexual life-history differences should be considered as a baseline scenario when attempting to understand how other factors (mate availability, sex differences in the costs of competing for mates) influence the evolution of parental care.

## Introduction

The evolution of parental care by males and females has been a central focus in evolutionary ecology (Trivers 1972; Baylis 1981; Clutton-Brock 1991; Queller 1997; Webb et al. 1999, 2002; Kokko and Jennions 2008; Alonzo 2010; Klug et al. 2012). Overall, maternal care is more prevalent than paternal care (Kokko and Jennions 2003, 2008), but there is large variation in which sex provides care both within and across animal groups (Zeh and Smith 1985; Clutton-Brock 1991; Beck 1998; Reynolds et al. 2002; Mank et al. 2005). Understanding such diverse patterns of parental care requires that we address two broad questions. First, it is important to understand what conditions lead to the origin of some pattern of care by males and/or females from an ancestral state of no care. Once some pattern of care (i.e., paternal, maternal, or bi-parental) is present in a system, however, one must further ask what life-history changes lead to transitions among different patterns of care. Parental care alters survival and reproduction of both parents and offspring. When care alters life-history characteristics such as survival and fecundity, the conditions that give rise to the origin of care will not necessarily be the same as those that favor transitions among different patterns of care. It is therefore important to examine separately the origin of and transitions among different sex-specific patterns of care. In our companion article (Klug et al. 2013), we identified the life-history conditions that most strongly select for the origin of care by males and/or females from an ancestral state of no care. In this study, we focus on transitions among paternal, maternal, and bi-parental care. Specifically, we envision the scenario in which care has originated and then assume that life-history traits of males and females may change either due to care itself (i.e., costs of caring such as decreased survival and future reproduction) or changes in unrelated factors (e.g., sex differences in predation, disease, competition, and/or maturation) that affect life-history traits such as survival and maturation. We then ask, given these changes, do transitions among maternal, paternal, or bi-parental care arise?

Transitions among different patterns of care have been relatively common in the evolutionary history of many animal groups. In ray-finned fishes, transitions among maternal, paternal, and bi-parental care occurred up to nine times (Mank et al. 2005). In anurans, there have been two transitions from paternal care to bi-parental care, up to two transitions from bi-parental to maternal care, and up to three transitions between maternal and paternal care (Beck 1998; Summers et al. 1999; Clough and Summers 2000; Reynolds et al. 2002). In primates, there have been 17–23 transitions from maternal to bi-parental care and 3–8 transitions from bi-parental to maternal care (Reynolds et al. 2002). Similarly, there have been up to three transitions from maternal to bi-parental care in crocodiles (Reynolds et al. 2002). In shorebirds, the most common evolutionary transition is from paternal to bi-parental care (5–11 transitions), but there have also been numerous transitions from paternal to maternal care (6–8 transitions) and from bi-parental to paternal care (2–6 transitions) (Székely and Reynolds 1995; Reynolds et al. 2002; see related analyses in Tullberg et al. 2002). In other birds, the majority of transitions have been from bi-parental to maternal care (7 transitions). There have also been two transitions from male to bi-parental care and two transitions from bi-parental care to paternal care, and one transition from paternal to maternal care (Reynolds et al. 2002). The prevalence of such transitions across taxonomic groups suggests that understanding sex differences in parental care requires that we understand why such transitions arise.

Numerous factors can influence transitions among different patterns of care. For example, transitions might be selected for if the benefits and/or costs of care vary between the sexes (Trivers 1972; Queller 1997; Royle et al. 2002; Kokko and Jennions 2008; Olson et al. 2009; Stiver and Alonzo 2009; Alonzo 2010). Such sex differences in benefits and costs of parental care can occur if males and females vary in their likelihood of re-mating (Trivers 1972; Queller 1997; Kokko and Jennions 2008) or certainty of parentage (Maynard Smith 1978; Baylis 1981; Winkler 1987; Westneat and Sherman 1993; Queller 1997; Sheldon 2002; Kokko and Jennions 2008; Alonzo 2010, 2012). In some animals, there are potentially physiological constraints on which sex can provide care that lead to sex-role specialization. In placental mammals, for instance, a loss of maternal care would be difficult because offspring depend upon maternal care for gestation and lactation. Offspring need can also affect transitions between uni- and bi-parental care. For example, if offspring need becomes very high, care from both parents might be necessary to ensure survival and this might select for bi-parental care (Thomas and Székely 2005; Székely et al. 2007). In addition to the factors above, males and females often differ in general life-history characteristics – that is, stage-specific survival and maturation rates – due to differences between the sexes in physiology, predation risk, costs and benefits of mating, and/or resource use. Such differences can in turn affect selection on parental care by males and females (Klug and Bonsall 2010; Bonsall and Klug 2011a,b).

Life-history differences between males and females in mortality and maturation (i.e., the process of becoming sexually mature) can be related to anisogamy (Trivers 1972), effects of sex hormones on development (Sockman and Schwabl 2000; Eising et al. 2001; Cook and Monaghan 2003), the costs of providing care if one sex provides care, and differences between the sexes in costs of mating. In our previous work, we found that sex differences in survival and maturation can favor the origin of maternal or paternal care, but anisogamy alone does not explain the prevalence of maternal care (Klug et al. 2013). How such life-history differences between males and females affect transitions among different patterns care is unknown. In this study, we use a general mathematical model to identify key life-history conditions that favor transitions among paternal, maternal, and bi-parental care. In the model, we assume that some form of care has originated. We first ask whether certain transitions will be favored when males and females are relatively similar. We then assume that life-history differences between the sexes arise (due to care or some other factor) and identify the differences between males and females that are likely to favor transitions among different patterns of care.

## Methods

We use a mathematical modeling approach (Metz et al. 1992; Dieckmann and Law 1996; Vincent and Brown 2005; Otto and Day 2007) to identify the life-history conditions that favor transitions among different patterns of care. The general modeling framework is identical to that of our companion article (Klug et al. 2013; see also Klug and Bonsall 2007; Klug and Bonsall 2010; Bonsall and Klug 2011a,b), but in this study, we consider cases in which some form of care is the ancestral state (in Klug et al. 2013 no care is always the ancestral state). In our modeling framework, we allow a rare mutant that exhibits paternal, maternal, or bi-parental care to invade a resident population in which a different form of parental care (paternal, maternal, or bi-parental) is already present and at equilibrium in the population. The alternative, mutant parental care strategy invades from rare into the population (as is standard in invasion analyses, Otto and Day 2007; eqn. 3–4). We assume a stage-structured system in which individuals pass through egg and juvenile stages and then mature and reproduce as adults. Mutant and resident individuals experience equivalent demographic processes (i.e., both residents and mutants have the same death, maturation, and reproductive rates before costs and benefits of care are accounted for). Parental care is then associated with benefits to offspring (increased offspring survival) and costs to the parent providing it (decreased parental survival; described below). Our approach differs from previous models focused on sex differences in parental care in a number of key ways. First, we assume that females are the limiting sex (described below), but beyond that, we do not explicitly focus on how sex differences in mate competition influence parental care, a major focus in many recent models of parental care. Instead, we focus primarily on how sex differences in basic life history can shape patterns of care. Additionally, while many models of care focus primarily on the dynamics of a single life-history stage, we explicitly consider how dynamics at multiple life-history stages can influence patterns of parental care.

### Model dynamics

The basic modeling dynamics below are identical to those of Klug et al. (2013). Males and females pass through egg (*E*) and juvenile stages and mature and reproduce as adults (*A*). Eggs decrease as they die and mature and increase as adults reproduce, such that


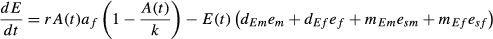
(1)

where *e*_*m*_ is the rate at which male eggs are produced and *e*_*f*_ is the rate at which female eggs are produced at time *t* (*e*_*m*_ = *e_f_* = 0.5 initially in all cases considered). Male and female eggs die at rates *d*_*Em*_ and *d*_*Ef*_. The rate of male eggs surviving the egg stage, *e*_*sm*_, equals 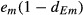. Likewise, the rate of female eggs surviving the egg stage, *e*_*sf*_, equals 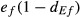. Those surviving male and female eggs then mature at rates *m*_*Em*_ and *m*_*Ef*_. Female fecundity limits reproduction (Bateman 1948) and reproduction in the population is assumed to be density-dependent. On average, each female produces *r* eggs that are fertilized. The total number of eggs that are fertilized is a function of *r*, the number of adults present *A(t)*, the rate at which females enter the adult stage *a*_*f*_, and the carrying capacity of the population *K*. The rate at which females enter the adult stage at time *t*, *a*_*f*_, equals 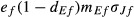, where *σ*_*Jf*_ represents female juvenile survival. Each fertilized egg has one mother and one father, and thus our measure of per capita female fecundity, *r*, is also a measure of the rate of egg fertilization in the population.

Adults in the population increase as individuals pass through the juvenile stage and decrease as adults die:


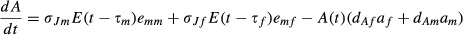
(2)

where *σ*_*Jm*_ and *σ*_*Jf*_ represent the juvenile survival rates of males and females, *e*_*mm*_ and *e*_*mf*_ are the rate of male and female eggs surviving the egg stage and maturing into juveniles, *τ*_*m*_ and *τ*_*f*_ are the durations of the male and female juvenile stages, and *d*_*Am*_ and *d*_*Af*_ are the rates at which male and female adults die. The rate at which males and females that survive the egg stage and mature into juveniles at time *t*, *e*_*mm*_ *and e*_*mf,*_ equals 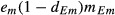 and 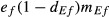. The adults that are male and female at time *t* is a function of the rate of individuals surviving the egg stage, maturing, and surviving and passing through the juvenile stage. Specifically, the rate at which males and females enter the adult stage at time *t*, *a*_*m*_ *and a*_*f,*_ equals 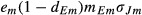 and 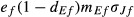.

The density of resident adults at equilibrium (i.e., when 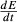 and 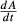 equal zero) is


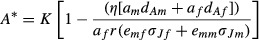
(3)

where *η* = *e*_*sf*_*m*_*Ef*_ + *e*_*sm*_*m*_*Em*_ + *e*_*f*_*d*_*Ef*_ + *e*_*m*_*d*_*Em*_. The dynamics of the rare mutant that provides parental care are given by the following equations and by incorporating the relevant trade-offs associated with parental care into the mutant and resident dynamics (discussed below and in Table 1). The other parameters are as described previously and superscript ^•^ denotes the new mutant strategy that exhibits parental care:


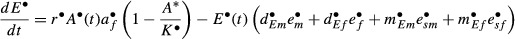
(4)


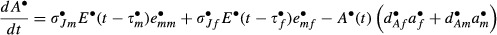
(5)

where A* (eqn. 3) is the equilibrial abundance of the resident adult population. As the mutant is assumed to be rare in the population, density-dependence operating on adult mutant reproduction occurs through competition with the resident (as is standard for ecological and evolutionary invasion analyses).

**Table 1:** Costs and benefits of initial investment in eggs by females 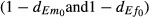 and parental care by males and females (*c*_*m*_ and *c*_*f*_). The total level of parental care provided to eggs, *c*_*total*_, is the sum of care provided by their mother and father, that is, *c*_*m*_ + *c*_*f*_. Male and female egg death rate decreases as initial investment in eggs increases and as the total level of parental care increases. Initial egg investment is assumed to be costly to mothers, such that female adult death rate increases and fecundity decreases as initial egg investment increases. Care is costly to parents, and as care increases, adult death rate also increases. The term *a* determines the specific shape of the trade-off function and is equal to 6 in all cases considered

	Trade-offs associated with parental care by males and/or females:	Example of trade-off:
Egg death rate (*d*_*Em*_ *& d*_*Ef*_)	Egg death rate ↓ as care ↑ *♂s*: 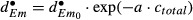 ♀s: 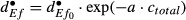	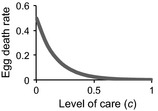
Adult death rate (*d*_*Am*_ *& d*_*Af*_)	Male adult death rate ↑ as care ↑ and Female adult death rate ↑ as initial egg investment ↑ and as care ↑ *♂s*: 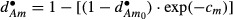 ♀s: 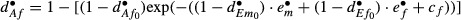	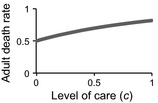
Female fecundity (*r*)	Female fecundity ↓ as initial egg investment ↑, i.e., Female fecundity ↓ as egg death rate in the absence of care ↓ 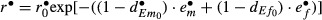	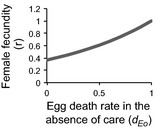

To consider transitions among different patterns of parental care, we consider the following scenarios: (1) paternal care invading maternal care; (2) maternal care invading paternal care; (3) maternal care invading bi-parental care; (4) bi-parental care invading maternal care; (5) paternal care invading bi-parental care; and (6) bi-parental care invading paternal care. For all scenarios (described further below), we identify the stage-specific maturation and mortality rates of mutant and resident males and females that will favor the invasion of the rare mutant strategy. In doing so, we identify the general life-history conditions expected to favor transitions among the different patterns of parental care.

In all cases, we assume that parents are associated with their offspring (due to spatial clumping or kin recognition) and remain alive long enough to provide care to young. Furthermore, the model assumes that at least a single male and single female of each strategy remain alive, and the parameter values considered never result in complete mortality of all of one sex.

### Costs and Benefits of Parental Care and Initial Egg Investment

Parents can affect offspring survival and quality by investing resources into eggs (referred to herein as initial egg investment) and providing post-fertilization parental care behavior (referred to as parental care) to offspring (see also Klug and Bonsall 2010). Here, we assume that females initially allocate resources to eggs, and male, female, or both male and female mutant parents can provide care to their eggs. For simplicity, and because egg care is more prevalent than juvenile care, we focus on parental care of developing zygotes and assume that juveniles do not receive care. The costs and benefits described below are identical to those assumed in our companion article, Klug et al. (2013), with the exception being that individuals exhibiting the resident strategy provide either paternal, maternal, or bi-parental care in the current model analyses (in Klug et al. 2013, no care is always the ancestral state).

Baseline egg death rate (i.e., egg death rate in the absence of any care) is used as our proxy of initial egg investment. Specifically, we assume that egg survival increases as initial egg investment increases. Initial egg investment is costly to females, such that as initial egg investment increases, female survival and fecundity decrease (Table 1). Specifically, this assumes that an increase in individual egg size is associated with an increase in total investment within a given reproductive bout. Importantly, because this assumption is unchanged across all of our scenarios, this basic assumption is unlikely to affect our general patterns. Parental care, which again is provided by mutant parent(s) to their mutant eggs and by the resident parent(s) to their resident eggs, increases egg survival, and the total level of care that eggs receive is the sum of the care provided by their male and female parents (*c*_*m*_ + *c*_*f*_) (Table 1). The benefits of care and of initial egg investment are additive, such that overall egg survival increases as initial egg investment and care increase (Table 1). Providing care is costly to the parent providing it, and as the level of care increases, adult survival declines (i.e., male and/or female death rate increases) (Table 1). As mentioned above, we assume that mutant and resident adult parents are able to provide care for their young.

In all cases, we assume asymptotic non-linear trade-offs (Table 1; these are identical to those of Klug et al. unpubl., ms.). These trade-offs allow us to consider all biologically realistic parameter values (death and maturation rates between zero and one). Non-linear trade-off functions are likely to be biologically realistic in many animals, as the benefits of care are typically thought to be diminishing (Clutton-Brock 1991), and our general patterns will hold for other similarly shaped functions.

The trade-offs described in Table 1 provide some insight into whether one form of parental care will lead to increased reproductive success (i.e., higher egg survival) in comparison with an alternative form of parental care. However, the costs and benefits associated with each pattern of care alone do not provide information on whether one pattern of parental care will be able to invade an alternative pattern of care and persist given the stage-structured life-history conditions and the ecological dynamics. Information on invasion of care necessitates further analysis and is described below. These invasion analyses allow us to ask whether paternal, maternal, and/or bi-parental care can invade an ancestral state of a different pattern of care given a set of specified male and female life-history parameters. This, in turn, allows us to identify the male and female life-history characteristics (stage-specific mortality and maturation) that are most likely to promote transitions among paternal, maternal, and/or bi-parental care.

### Fitness of Parental Care & Invasion Dynamics

The fitness of the rare mutant that provides parental care is the per capita population-level growth rate and this is found by taking the determinant of the invasion matrix:


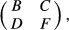
(6)

Where


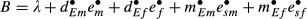
(7)


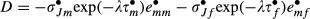
(8)


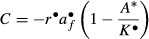
(9)


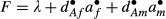
(10)

and solving the resulting characteristic equation for λ (i.e., the fitness of the mutant strategy relative to that of the resident; see also Metz et al. 1992 and Vincent and Brown 2005) when selection is relatively weak (λ is small such that exp(− *λτ*) ≈ (1− *λτ*)).

In all cases, we assume that baseline conditions (i.e., those before any costs and benefits of care are accounted for) are identical for the mutant and resident strategy. We then determine the fitness of the mutant strategy (paternal, maternal, or bi-parental care) relative to that of a different resident strategy (paternal, maternal, or bi-parental care) in relation to varying male and female life-history parameters. We then ask under what conditions each pattern of care will invade the other patterns of care. We do this for varying male and female egg mortality, egg maturation rate, juvenile survival, duration of the juvenile period, and adult mortality. This allows us to ask whether transitions among different patterns of parental care occur due to life-history differences between males and females.

## Results

### Transitions to maternal care are favored when males and females are relatively similar

**Figure 1:**
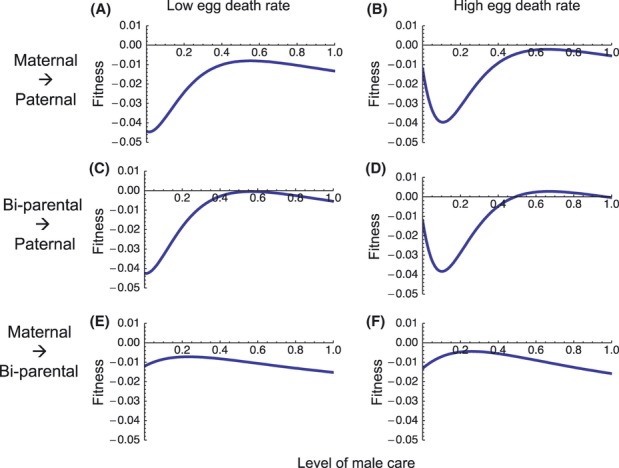
Transitions to increased male care are rare when males and females have similar life-history characteristics. For various levels of male care, we show the fitness gain associated with (A–B) paternal care relative to an ancestral state of maternal care (*c*_*f*_ *= 0.7*), (C–D) paternal care relative to an ancestral state of bi-parental care (*c*_*m*_ *= 0.35, c*_*f*_ *= 0.35*), and (E–F) bi-parental care (where *c*_*f*_ *= 0.35*) relative to an ancestral state of maternal care (*c*_*f*_ *= 0.7*). These transitions are shown when (A, C, E) egg death rate in the absence of care is relatively low (i.e., females have high initial egg allocation; *d*_*E*__m0_ = *d*_*E*__*f0*_ = 0.5) and (B, D, F) egg death rate in the absence of care is high (i.e., females have low initial egg allocation; *d*_*E*__m0_ = *d*_*E*__*f0*_ = 0.9). Unless otherwise noted, *m*_*E*__m_ = *m*_*E*__f_ = 0.5, *r*_*0*_ = 6, *d*_*A*__*m0*_ = *d*_*A*__*f0*_ = 0.5, *K* = 50, *σ*_*Jm0*_ = *σ*_*Jf0*_ = 0.5, *τ*_*m*_ = *τ*_*f*_ = 0.1, *e*_*m*_ = *e*_*f*_ = 0.5 for both residents and mutants.

**Figure 2:**
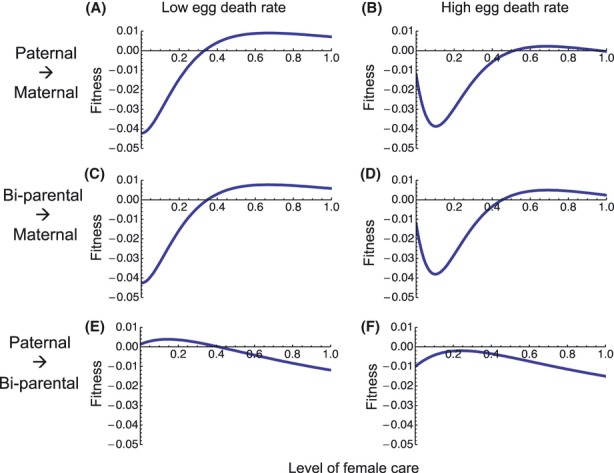
Transitions to maternal care will favor relatively high levels of female care, whereas transitions to bi-parental care will favor relatively low levels of female care when males and females have similar life-history characteristics. For various levels of female care, we show the fitness gain associated with (A–B) maternal care relative to an ancestral state of paternal care (*c*_*m*_ *= 0.7*), (C–D) maternal care relative to an ancestral state of bi-parental care (*c*_*m*_ *= 0.35, c*_*f*_ *= 0.35*), and (E–F) bi-parental care (where *c*_*m*_ *= 0.35*) relative to an ancestral state of paternal care (*c*_*m*_ *= 0.7*). These transitions are shown when (A, C, E) egg death rate in the absence of care is relatively low (i.e., females have high initial egg allocation; *d*_*E*__m0_ = *d*_*E*__*f0*_ = 0.5) and (B, D, F) egg death rate in the absence of care is high (i.e., females have low initial egg allocation; *d*_*E*__m0_ = *d*_*E*__*f0*_ = 0.9). Unless otherwise noted, *m*_*E*__m_ = *m*_*E*__f_ = 0.5, *r*_*0*_ = 6, *d*_*A*__m0_ = *d*_*A*__*f0*_ = 0.5, *K* = 50, *σ*_*Jm0*_ = *σ*_*Jf0*_ = 0.5, *τ*_*m*_ = *τ*_*f*_ = 0.1, *e*_*m*_ = *e*_*f*_ = 0.5 for both residents and mutants.

Evolutionary transitions that result in increased paternal care (maternal → paternal, bi-parental → paternal, maternal → bi-parental) are unlikely when males and females have similar life-history characteristics (i.e., similar mortality, similar maturation, and similar investment in gametes; Fig. 1). The exception to this pattern is when bi-parental care is the ancestral state and baseline egg death (i.e., egg death rate prior to any care) is relatively high. Under these conditions, moderate to high levels of male-only care will be favored by selection (Fig. 1D). In contrast, transitions to increased maternal care (paternal → maternal, bi-parental → maternal, paternal → bi-parental) are more likely (Fig. 2). This is particularly true when egg death rate in the absence of care is relatively low (Fig. 2 A, C, E). When egg death rate in the absence of care is relatively low, females have already invested substantially in eggs and there are greater inherent differences between males and females (i.e., when females invest heavily into eggs, they have higher mortality than males independent of any care that is provided). Relatively high female egg allocation means that females have reduced future reproduction and survival. As a result, selection favors increased investment by females in current reproduction (Fig. 2).

### Sex differences favor transitions among paternal, maternal and bi-parental care

**Figure 3:**
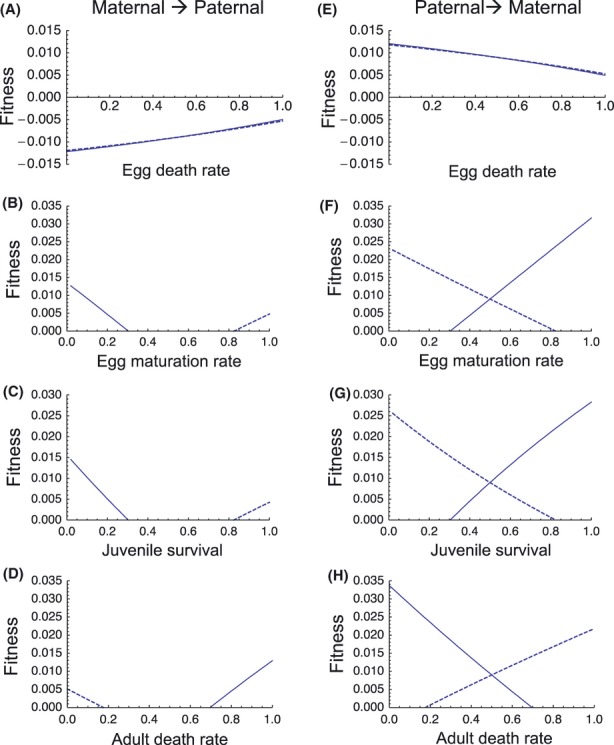
Life-history differences between the sexes favor evolutionary transitions between maternal and paternal care. For several life-history characteristics of males (solid line) and females (dashed line), we show the fitness gain associated with (A–D) paternal care (*c*_*m*_ *= 0.7, c*_*f*_ *= 0*) relative to an ancestral state of maternal care (*c*_*m*_ *= 0, c*_*f*_ *= 0.7*) and (E–H) maternal care (*c*_*m*_ *= 0, c*_*f*_ *= 0.7*) relative to an ancestral state of paternal care (*c*_*m*_ *= 0.7, c*_*f*_ *= 0*). Transitions from maternal to paternal care will be most likely when (A) egg death rate in the absence of care is high, (B) male eggs mature slowly and female eggs mature quickly, (C) male juvenile survival is low and female juvenile survival is high, and (D) male adult death rate is high and female adult death rate is low. In contrast, maternal care is most likely to arise from a state of paternal care when (E) egg death rate in the absence of care is low, (F) male eggs mature quickly and female eggs mature slowly, (G) male juvenile survival is high and female juvenile survival is low, and (H) male adult death rate is low and female adult death rate is high. Unless otherwise noted, *d*_*E*__m0_ = *d*_*E*__*f0*_ = 0.5, *m*_*E*__m_ = *m*_*E*__f_ = 0.5, *r*_*0*_ = 6, *d*_*A*__m0_ = *d*_*A*__*f0*_ = 0.5, *K* = 50, *σ*_*Jm0*_ = *σ*_*Jf0*_ = 0.5, *τ*_*m*_ = *τ*_*f*_ = 0.1, *e*_*m*_ = *e*_*f*_ = 0.5 for both residents and mutants. A single line indicates that the relationships between fitness and male and female egg death rates are indistinguishable (i.e., the individual lines overlap).

**Figure 4:**
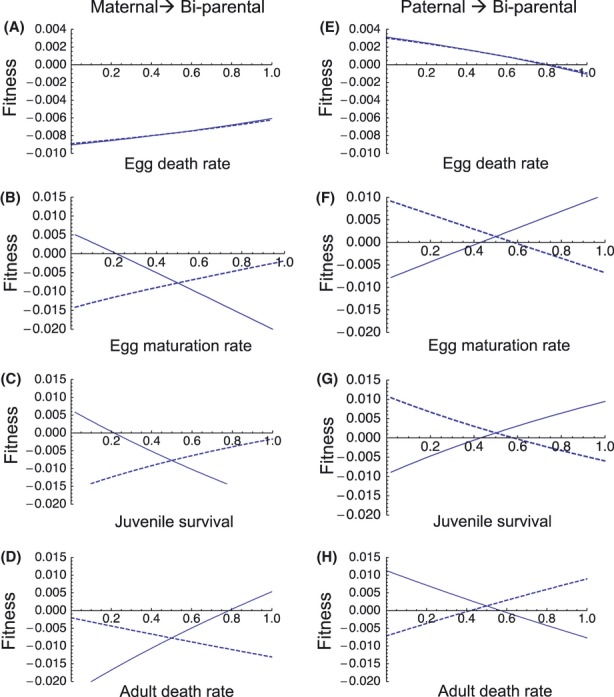
Life-history differences between the sexes favor evolutionary transitions from uni-parental to bi-parental care. For several life-history characteristics of males (solid line) and females (dashed line), we show the fitness gain associated with (A–D) bi-parental care (*c*_*m*_ *= 0.35, c*_*f*_ *= 0.35*) relative to an ancestral state of maternal care (*c*_*m*_ *= 0, c*_*f*_ *= 0.7*) and (E–H) bi-parental care (*c*_*m*_ *= 0.35, c*_*f*_ *= 0.35*) relative to an ancestral state of paternal care (*c*_*m*_ *= 0.7, c*_*f*_ *= 0*). The conditions that give to a transition from maternal to bi-parental care are identical to those that give rise to a transition from maternal to paternal care (Fig. 3). Likewise, the conditions that give rise to a transition from paternal to bi-parental care are identical to those that give rise to a transition from paternal to maternal care (Fig. 3). Unless otherwise noted, *d*_*E*__m0_ = *d*_*E*__*f0*_ = 0.5, *m*_*E*__m_ = *m*_*E*__f_ = 0.5, *r*_*0*_ = 6, *d*_*A*__*m0*_ = *d*_*A*__*f0*_ = 0.5, *K* = 50, *σ*_*Jm0*_ = *σ*_*Jf0*_ = 0.5, *τ*_*m*_ = *τ*_*f*_ = 0.1, *e*_*m*_ = *e*_*f*_ = 0.5 for both residents and mutants. A single line indicates that the relationships between fitness and male and female egg death rates are indistinguishable (i.e., the individual lines overlap).

**Figure 5:**
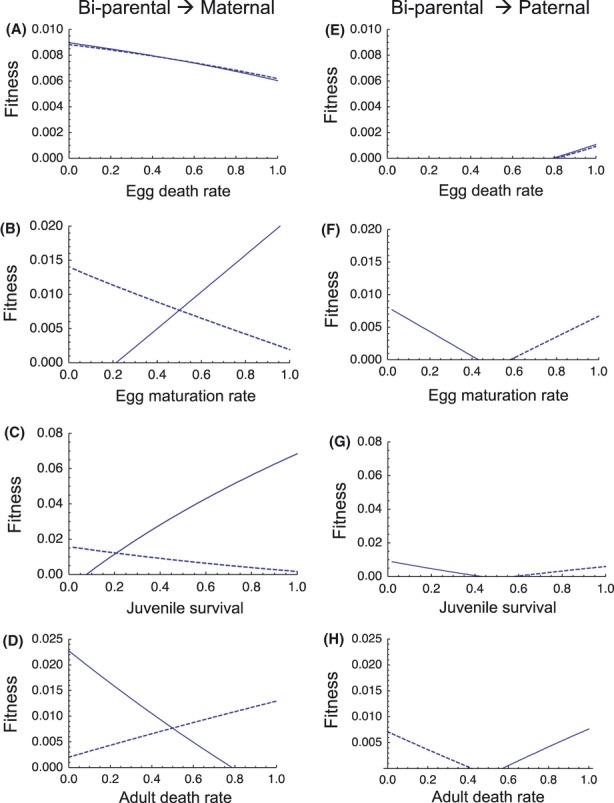
Life-history differences between the sexes favor evolutionary transitions from bi-parental to uni-parental care. For several life-history characteristics of males (solid line) and females (dashed line), we show the fitness gain associated with (A–D) maternal care (*c*_*m*_ *= 0, c*_*f*_ *= 0.7*) relative to an ancestral state of bi-parental care (*c*_*m*_ *= 0.35, c*_*f*_ *=0.35*) and (E-H) paternal care (*c*_*m*_ *= 0.7, c*_*f*_ *= 0*) relative to an ancestral state of bi-parental care (*c*_*m*_ *= 0.7, c*_*f*_ *= 0*). The conditions that give to a transition from bi-parental to maternal care are identical to those that give rise to a transition from paternal to maternal care (Fig. 3). Likewise, the conditions that give rise to a transition from bi-parental care to paternal care are identical to those that give rise to a transition from maternal to paternal care (Fig. 3). Unless otherwise noted, *d*_*E*__m0_ = *d*_*E*__*f0*_ = 0.5, *m*_*E*__m_ = *m*_*E*__f_ = 0.5, *r*_*0*_ = 6, *d*_*A*__*m0*_ = *d*_*A*__*f0*_ = 0.5, *K* = 50, *σ*_*Jm0*_ = *σ*_*Jf0*_ = 0.5, *τ*_*m*_ = *τ*_*f*_ = 0.1, *e*_*m*_ = *e*_*f*_ = 0.5 for both residents and mutants. A single line indicates that the relationships between fitness and male and female egg death rates are indistinguishable (i.e., the individual lines overlap).

**Figure 6:**
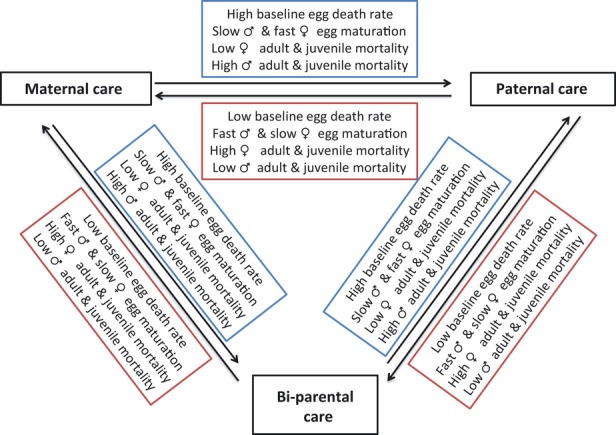
Life-history differences between males and females favor transitions among maternal, bi-parental, and paternal care. Transitions to increased male care (maternal → paternal, bi-parental → paternal, maternal → bi-parental; blue boxes) are favored when egg death rate in the absence of care is high, male egg maturation is slow and female egg maturation is high, female adult and juvenile mortality is low, and male adult and juvenile mortality is high. Transitions to increased female care (paternal → maternal, bi-parental → maternal, paternal → bi-parental; red boxes) are favored when egg death rate in the absence of care is low, male egg maturation is fast and female egg maturation is slow, female adult and juvenile mortality is high, and male adult and juvenile mortality is low.

As mentioned above, males and females will often differ in mortality and maturation due to factors unrelated to care, such as sex differences in predation rate, resource use, costs of mating, and physiology, and such differences can favor transitions in care. In general, transitions to increased maternal care are more likely to be selected for than those associated with increased paternal care (Figs. 1–5). However, there are specific combinations of male and female life-history characteristics that favor transitions to increased paternal care (Fig. 6). A transition from maternal to paternal care will be most strongly favored when (1) egg death rate in the absence of care is high (Fig. 3A); (2) male eggs mature slowly and female eggs mature quickly (Fig. 3B); (3) male juvenile survival is low and female juvenile survival is high (Fig. 3C); and (4) male adult death rate is high and female adult death rate is low (Fig. 3D). When males experience higher death rates and mature more slowly (which increases their likelihood of dying before they mature and leads to them being older at maturation), they have reduced potential for future reproduction, and this is likely why males are more likely to invest more in current reproduction (i.e., care) under these conditions. Transitions from maternal to bi-parental and from bi-parental to paternal care will be favored under the same conditions (Fig. 4A–D, Fig. 5E–H, Fig. 6). In other words, the life-history conditions that give rise to increased paternal investment are qualitatively the same, regardless of the specific transition being considered (i.e., maternal → paternal, bi-parental → paternal or maternal → bi-parental). If we consider the magnitude of the fitness gain associated with each of these three transitions for given life-history parameter values, the transition from maternal to paternal care results in the highest fitness (Fig. 3A–C vs. Fig 4A–D and Fig. 5E–H).

Life-history differences between males and females can also favor increased maternal care (Fig. 6). Transitions from paternal to maternal, paternal to bi-parental, and bi-parental to maternal will result in the greatest fitness gains when 1) egg death rate in the absence of care is low (Figs. 4 E and Fig. 5A); (2) male eggs mature quickly and female eggs mature slowly (Figs. 4 F and Fig. 5B); (3) male juvenile survival is high and female juvenile survival is low (Figs. 4 G and Fig. 5C); and (4) male adult death rate is low and female adult death rate is high (Figs. 4 H and Fig. 5D). When females have higher mortality and mature more slowly, they have reduced future expected reproductive success, and this favors increased investment in current reproduction. For a given set of life-history values, the transition from paternal to maternal care is the transition resulting in increased maternal care that results in highest fitness gains (Fig. 3E–H vs. Fig 4E–H and Fig. 5A–D).

### If each sex can effectively provide care, transitions to bi-parental care are rare when the total level of care is held constant for all patterns of care

If the ancestral state is maternal care, transitions to paternal care will be more likely than those to bi-parental care, all else being equal (Fig. 3A–D vs. Fig 4A–D). Similarly, if the ancestral state is paternal, transitions to maternal care will result in greater fitness (i.e., net benefits in lifetime reproductive success) than transitions to bi-parental care, all else being equal (Fig. 3E–H vs. Fig 4E–H). This suggests that simple life-history differences between the sexes are alone unlikely to explain transitions to bi-parental care if the level of care provided by both parents is equal to the level of care provided by an individual parent. In other words, if a single parent can provide sufficient care alone, bi-parental care that does not increase the overall level of care is unlikely to arise. If bi-parental care is the ancestral state, transitions to maternal care tend to be associated with higher fitness than transitions to paternal care (Fig. 5). In particular, transitions from bi-parental care to maternal care will be strongly favored when egg mortality is low, male eggs mature quickly and female eggs mature slowly, male juveniles have high survival and female juveniles have low survival, and adult mortality is high for females and low for males (Fig. 5A–D). In contrast, transitions to paternal care will be favored when egg death mortality is high, male eggs mature slowly and female eggs mature quickly, male juvenile survival is low and female juvenile survival is high, and male adult mortality is high and female adult mortality is low (Fig. 5E–H).

## Discussion

Evolutionary transitions among paternal, maternal, and bi-parental care are unlikely to be favored when males and females are relatively similar – that is, when baseline mortality and maturation rates are similar for both sexes, initial egg investment by females is relatively small, and low levels of care are provided by one or both sexes (Figs. 2 B, D & F). In other words, when males and females are similar, whatever pattern of care originated in a population is likely to be maintained.

As males and females become more dissimilar, evolutionary transitions become more likely. If females and males differ substantially because females invest heavily into eggs initially (i.e., egg death rate is low even in the absence of care), transitions to maternal care are expected if females provide moderate to high levels of care (Fig. 2 A, C). Overall, this means that transitions to increased maternal care (paternal → bi-parental, paternal → maternal, or bi-parental → maternal) tend to result in greater fitness gains (i.e., greater lifetime reproductive success) than transitions to increased paternal care (bi-parental → paternal, maternal → paternal, or maternal → bi-parental) (Fig. 1A–F vs. Fig. 2 A–F).

The pattern of transitions to maternal care being favored is consistent with empirical work demonstrating the prevalence of maternal care in animals (reviewed in Kokko and Jennions 2008), previous evidence of transitions to maternal care in nature (e.g., Reynolds et al. 2002), and some previous theory suggesting that anisogamy causes females to have reduced survival and future reproductive opportunities, which in turn selects for them to invest more in current (rather than future) reproduction (Sargent and Gross 1985; see also related discussion in Kokko and Jennions 2008). This finding, however, is inconsistent with some other theoretical work. Kokko and Jennions (2008) found that anisogamy is, in general, not sufficient to explain differences in maternal and paternal care and that the relative costs of competing for mates and caring for young can have strong influences on patterns of care. Our modeling framework differs from that of previous models in several ways, and this might explain why some of our results differ from previous work. First, many recent models on parental care focus on the role that sexual selection plays in explaining patterns of care. Although we assume that females are the limiting sex (a single male can fertilize the eggs of all females), we make no additional assumptions about sexual selection and instead focus more explicitly on how life-history characteristics at multiple life-history stages can influence the benefits of care. In this sense, our framework can be thought of as a baseline or null scenario: we ask whether life-history similarities or differences between the sexes can alone lead to transitions among care even in the absence of more complex assumptions about mate competition and choice.

Our previous work focused on the origin of maternal, paternal, and bi-parental care from an ancestral state of no care suggests that anisogamy alone does not favor the origin of maternal care over paternal care (Fig. 1 of Klug et al. 2013). The differences between these predictions suggest that the evolutionary conditions favoring the origin of different patterns of care are not necessarily the same as the factors that favor transitions among care patterns. Such differences between the origin of and transitions among different patterns of care can be related to the differences between males and females that arise from one sex providing care and/or increased offspring survival if some pattern of care is present. For example, if one parent already provides care, this is likely to be costly and reduce future survival and reproduction in comparison with the other parent who does not provide care. Our findings suggest that such differences between parents due to care can affect transitions among different forms of care. Additionally, differences in the strength of selection between the origin of and transitions among care might cause sex differences in initial gametic investment (which lead to sex differences in expected future survival and reproduction) to have greater influence on transitions among care.

As described above, the fitness gains associated with transitions among different patterns of care tend to be relatively small when males and females are similar (i.e., the fitness gains tend to be less than those associated with the origin of care under the conditions considered, Klug et al. 2013). Thus, it is possible that if the degree of anisogamy increases once some male care is already present, relatively weak selection can favor increased maternal care. In anurans, birds, and cichlid fishes, transitions to increased maternal care tend to be more common than those to increased paternal care. In anurans, there have been up to six transitions leading to increased maternal care (paternal → bi-parental, paternal → maternal, or bi-parental → maternal) and only zero to two transitions to increased paternal care (bi-parental → paternal, maternal → paternal, or maternal → bi-parental) (Reynolds et al. 2002). This pattern is consistent with our finding that evolutionary transitions to increased maternal care will be more common than transitions to increased paternal care when some pattern of care is already present in a system. However, it remains unclear whether anisogamy, increased female mortality, or reduced female maturation rates played a role in these transitions to maternal care. This would certainly be an interesting question to address in a future comparative study focused on examining the relationship between sex-specific mortality and maturation during different life-history stages and transitions among care.

In contrast to the above pattern in anurans, birds, and cichlid fishes, transitions to increased paternal care have occurred more frequently than transitions to increased maternal care in primates and crocodilians (Reynolds et al. 2002). In ray-finned fishes, there have been similar numbers of transitions leading to increased maternal care and increased paternal care (Mank et al. 2005). Previous work has linked these transitions to fertilization mode (Mank et al. 2005) and offspring need (Thomas and Székely 2005; Székely et al. 2007). Our findings suggest that such transitions can also be favored by life-history differences between males and females.

A variety of transitions will be selected for if males and female differ in life-history characteristics (Fig. 6). Our model suggests that transitions to increased paternal care (bi-parental → paternal, maternal → paternal, or maternal → bi-parental) will be favored when baseline male adult mortality is high, whereas transitions to increased maternal care (bi-parental → maternal, paternal → maternal, or paternal → bi-parental) will be favored when female adult mortality is high. When adult mortality is high in the absence of care, the costs of care will often be less than those when mortality is low because the mortality never exceeds one (i.e., the probability of dying at any given point in time is <1). Additionally, when adult mortality is high, individuals have reduced opportunities for future reproduction and are expected to invest more heavily in current reproduction (Stearns 1992; Tallamy and Brown 1999; Klug and Bonsall 2010). The finding that high adult mortality will favor parental care is consistent with the patterns on the evolutionary origins of care (Klug and Bonsall 2010). Furthermore, the finding that high adult mortality in one sex will favor parental care in that sex is also consistent with the patterns found when we focused on the origin of maternal and paternal care from an ancestral state of no care (Klug et al. 2013). This is potentially related to the idea that individuals experiencing high mortality have little opportunity for future reproduction and are therefore selected to invest more in current reproduction. Empirically, Winemiller and Rose (1992) found a relationship between short lifespan and the evolution of parental care in fishes. In addition to factors such as fertilization mode, offspring need, and differing costs of competing for mates versus caring for offspring, simple differences between males and females in mortality and maturation might have influenced the diversity of transitions seen in some animal groups, such as birds and fishes (e.g., Kokko and Jennions 2008).

Transitions to increased paternal care are also favored if egg death rate in the absence of care is high. The finding that care will be selected for when eggs need care the most is consistent with the previous work (Clutton-Brock 1991; Webb et al. 1999; Klug and Bonsall 2010; Bonsall and Klug 2011a,b; Klug et al. 2013). Our results focused on transitions to paternal care additionally suggest that paternal care in animals such as birds and fishes might be partially explained by high offspring need, and that high offspring need is more likely to explain the occurrence of paternal rather than maternal care, as transitions to paternal care were more likely when egg death rate in the absence of care is very high (i.e., when females have invested relatively little in eggs initially; Fig. 6). Indeed, we found that transitions to increased maternal care will be favored when egg death rate in the absence of care is relatively low. When egg death rate in the absence of care is low, females have invested heavily into eggs, and hence they have reduced future reproductive opportunities and might be selected to invest more heavily in current reproduction. This finding further highlights that the conditions that give rise to the origin of care from an ancestral state of no care can be different from those that favor transitions among different patterns of care.

When male eggs mature slowly, transitions to increased paternal care will be favored, and when male eggs mature quickly, transitions to increased maternal care will be favored. This pattern is consistent with the case in which we focused on the origin of different patterns of care from an ancestral state of no care (Klug et al. 2013). When slow-developing eggs reach maturity, they are older than fast-developing eggs, and therefore potentially have reduced future reproductive potential. This, in turn, might favor increased investment in any current reproduction once those males reach maturity. When female eggs mature slowly, transitions to increased maternal care will be favored, and when female eggs mature quickly, transitions to increased paternal care will be favored. When females mature relatively slowly, they are older when they mature in comparison with quickly maturing females, and this potentially leads to slow-developing females having reduced future reproductive potential and investing more in current reproduction. This is in contrast to the case in which we considered the origin of care. When focusing on the origin of care from an ancestral state of no care, relatively fast female egg maturation favors maternal, paternal, or bi-parental care equally (Klug et al. 2013). This qualitative difference further demonstrates how the conditions favoring the origin of care will often vary from those leading to transitions among different patterns of care. Whether offspring maturation rate plays a role in the origin of or transitions among different patterns of care is unknown in most animals and warrants further attention. In particular, egg maturation rate might have influenced the evolution of different patterns of care in anurans and birds. The evolution of larger eggs that presumably take longer to develop precedes the evolution of parental care in salamanders and frogs (Summers et al. 2006; see also Nussbaum 1985, 1987). Additionally, previous empirical work has found sex differences in development time in birds (Sockman and Schwabl 2000; Eising et al. 2001; Cook and Monaghan 2003) and other animals (Badyaev 2002). Whether sex differences in egg maturation rates exist in nature and influence transitions among care patterns still remains unknown.

In general, transitions to bi-parental care will be uncommon if one sex can provide sufficient levels of care. However, if one parent is not capable of providing enough parental care to ensure offspring survival, bi-parental care can be favored under some conditions (Figs. 1–6). In many animals, such as birds and some mammals, ensuring offspring survival likely requires care by both parents (Thomas and Székely 2005; Székely et al. 2007). High levels of offspring need and/or limits to a single parent's ability to provide sufficient care are likely to have played a strong role in the evolution of bi-parental care, and this is not considered in the current model. Additionally, in many species, each sex specializes on a particular form of care (e.g., lactation in female placental mammals). Sex-role specialization might also play a large role in determining which parent provides care, and this is also something that is not examined in the current modeling framework.

Previous work has shown that numerous factors can influence transitions among different patterns of parental care. Fertilization mode affects the evolution of paternal versus maternal care in fishes (Mank et al. 2005). Sexual selection and sexual conflict affect parental care in shorebirds (Parker et al. 2002; Thomas and Székely 2005; Székely et al. 2007; Olson et al. 2009) and cichlid fishes (Gonzalez-Voyer et al. 2008). Additionally, physical proximity to offspring (Williams 1975; Baylis 1981), costs and benefits of competing for mates versus caring (Kokko and Jennions 2008), and certainty of paternity (Trivers 1972; Kokko and Jennions 2008; Alonzo 2010) are expected to influence the evolution of care of by males and females. Offspring need and the amount of care provided by the other parent is expected to influence patterns of parental care (Parker et al. 2002). Thomas and Székely (2005) found that species with less demanding young are more likely to have uni-parental care than species with more demanding young. In addition to these factors, our work demonstrates that simple life-history differences between males and females in rates of mortality and/or maturation can drive transitions among paternal, maternal, and bi-parental care. The idea that life-history differences can alone lead to transitions among different patterns of care should be considered in the context of baseline expectation when examining whether other factors are responsible for evolutionary patterns of care. Additionally, future comparative studies could examine the relationship between sex-specific life-history characteristics and transitions among care. Our theoretical work provides testable predictions regarding the life-history characteristics that are most likely to favor transitions among maternal, paternal, and bi-parental care.
